# The role of different adjuvant therapies in locally advanced gastric adenocarcinoma

**DOI:** 10.18632/oncotarget.26106

**Published:** 2018-09-21

**Authors:** Ilaria Benevento, Nadia Bulzonetti, Francesca De Felice, Daniela Musio, Massimo Vergine, Vincenzo Tombolini

**Affiliations:** ^1^ Department of Radiotherapy, Policlinico Umberto I, Sapienza University of Rome, Rome 155, Italy; ^2^ Department of Surgical Sciences, Policlinico Umberto I, Sapienza University of Rome, Rome 155, Italy

**Keywords:** adjuvant therapy, chemoradiotherapy, radiotherapy, gastric cancer

## Abstract

**Background and Purpose:**

Complete surgical resection remains the only curative treatment option in locally advanced gastric cancer (GC). Several studies were conducted to prevent local recurrence and to increase the chance of cure. The aim of this study was to summarize our experience in locally advanced GC patients treated with adjuvant chemoradiotherapy (CRT) and to evaluate overall survival (OS), disease-free survival (DFS), toxicity rate and compliance to treatment.

**Materials and Methods:**

Locally advanced GC stage IB-III were included. Adjuvant CRT consisted of 45–50.4 Gy (1.8 Gy/day, 5 days/week) with concomitant Macdonald regimen (Mcd) or Epirubicin, Cisplatin and 5-Fluorouracil (ECF) scheme. Univariate and multivariate analysis of several prognostic factors for OS was conducted.

**Results:**

Fourty-nine GC patients were treated: 24 received Mcd and 25 received ECF. Median follow up was 48 months. Acute grade 3–4 toxicity was observed in 6 patients. The 2-year and 5-year OS rates were 65.3% and 41.5%, respectively. The 2-year and 5-year DFS were 59.2% and 41.2%, respectively. No prognostic factors were significantly associated with OS.

**Conclusions:**

Adjuvant CRT is a feasible strategy in locally advanced GC. It has an acceptable toxicity rate and it is able to increase both DFS and OS.

## INTRODUCTION

Complete surgical resection represents the only curative treatment option in locally advanced gastric cancer (GC). Clinical outcomes are still strongly influenced by the high percentage of both local recurrence (LR) and distant metastases rates. Approximately 60% of GC patients presented locally advanced disease at diagnosis. Although a complete tumor resection with negative margins (R0) can be achieved in 40–60% of these cases, about 70–90% of patients will subsequently relapse. An extended lymph node dissection might improve local control rate, but its optimal extent remains unresolved.

In order to reduce LR rate and increase cure rate of GC patients, several studies have tested different treatment strategies after surgery. US Intergroup Study INT-0116 [1] was the first large randomized trial that compared adjuvant chemoradiotherapy (CRT) versus surgery alone in patients with locally advanced disease. This study demonstrated a significant impact on progression free survival (PFS) and overall survival (OS), although mainly the benefit appeared to be derived from a reduction in LR rather than in distant metastases rate.

The aim of this study was to report our data concerning patients with locally advanced GC treated with adjuvant CRT, in order to analyze treatment compliance, toxicity rate and survival outcomes.

## MATERIALS AND METHODS

### Patient selection

Patients with locally advanced GC treated at our Department from 2000 to 2011 were retrospectively reviewed. All patients were assessed in a multidisciplinary clinic board by radiation oncologist, gastrointestinal surgeon and medical oncologist. The study was approved by the Institutional Reviewed Board and patients signed an informed consent.

Eligibility criteria included histologically confirmed gastric adenocarcinoma; stage IB-III disease; lack of involvement of esophagus; no distant metastases; a performance status of 0–2 according to the criteria of the Eastern Cooperative Oncology Group (ECOG); age greater than 18 years; adequate liver, renal and bone-marrow function.

### Treatment

All patients underwent surgery: gastrectomy or gastric resection with limited (D1) or extended (D2) lymph node dissection was performed at least 3 months before starting adjuvant CRT. Prior to CRT, all patients were subjected to post-surgical revaluation with clinical examination and total-body computed tomography scan.

All patients were treated with a concomitant adjuvant treatment.

Radiotherapy (RT) consisted of 45 Gy in 25 fractions (1.8 Gy/fraction, 5 days/week). In patients with resection margin microscopically involved (R1) the total dose was 50.4 Gy, with boost of 5.4 Gy.

From 2000 to 2004, external beam RT was delivered with a two-dimensional technique (2DRT) with anterior-posterior opposing fields and in the next years using three-dimensional RT (3DRT) with a multiple field technique.

The planning target volume (PTV) included tumor bed, as defined by preoperative imaging, residual stomach or anastomosis site with a safety margin of at least 2 cm and regional lymph nodes, based on the location of the primary tumor and the type of surgical procedure carried out. Regional lymph nodes included the perigastric, the periesophageal, the celiac axis, the para-aortic, the hepato-duodenal and pancreatic-duodenal lymph nodes as well as the nodes along the splenic artery to the splenic hilum and along the hepatic artery to the hepatic hilum. The dose planning for PTV and organs at risk (OAR) were performed according to the ICRU 50 [2] and 62 [3] guidelines.

A central venous access was placed for the administration of concomitant chemotherapy (CT). According to medical oncologist, two CT regimens were used: Macdonald scheme (Mcd) and Epirubicin, Cisplatin and 5-Fluorouracil scheme (ECF). Mcd consisted of continuous infusion of 5-Fluorouracil (5-FU, 425 mg/mq per day) plus Leucovorin (LV, 20 mg/mq per day) for 5 days, given every 3 weeks for two cycles, followed by continuous infusion of 5-FU (400/mg/mq/day) and LV (20 mg/mq/day) on days 1–4 and 23–25 during concomitant RT, and an additional cycles of 5-FU (425 mg/mq/day) and LV (20 mg/mq/day). The ECF scheme consisted of Epirubicin (50 mg/mq), Cisplatin (60 mg/mq) and 5-FU (200 mg/mq/day) before (1 cycle) and after (2 cycle) concurrent CRT.

### Toxicity

During CRT patients were evaluated daily. Toxicity was graded according to the Common Terminology Criteria for Adverse Events (NCI-CTCAE), version 4.0 [4].

### Follow-up

After adjuvant therapy, post-treatment surveillance was performed every 3 months for the first 2 years, every 6 months for the subsequent 3 years and then annually. Follow-up consisted of physical examination, a complete blood cell count, liver and renal function tests, level of tumour markers (CEA, Ca 19.9), abdominal ultrasound, total body computed tomography scan and PET/CT as clinically indicated.

### Statistical analysis

Standard descriptive statistics were used to evaluate the distribution of each factor. Continuous data were given as median (range), and categorical data as the number of observations and ratios.

OS and DFS were calculated in months from the date of the end of treatment to the first event, including date of the last follow-up or death (OS), and/or relapse (DFS).

OS and DFS were estimated using the Kaplan–Meier method and survival curves were compared using the log-rank test.

The following variables were investigated: sex (male versus female), age in years at diagnosis (< 65 versus ≥ 65), tumor grading (G1-2 versus G3), tumor site (cardia versus body-pylorus), CT regimen (Mcd versus ECF) and type of lymph node dissection (D1 versus D2). Variables associated with a *p*-value < 0.25 would be included in a multivariate survival analysis performed using Cox proportional hazard model. All reported *p* values are two-sided, and *p*-values lower than 0.05 were considered significant. Statistical analysis was performed using RStudio-0.98.1091 software.

## RESULTS

### Baseline characteristics

Between 2000 and 2011, 49 locally advanced GC patients were treated at our institution. Baseline patient and tumour characteristics are shown in Table 1. Median age was 59 years (35–78) and 23 patients (46.9%) were male. Median follow up was 48 months (3–129).

**Table 1:** Baseline patients' and tumours' characteristics

Patients' characteristic	*N* (%)	Tumours' characteristic	*N* (%)
Sex:		Adenocarcinoma	49 (100)
Male	23 (46.9)		
Female	26 (53.1)		
Age:		Grading:	
Median	59	G2	12 (24.5)
Range	35–78	G3	35 (71.4)
		G4	2 (4.1)
PS ECOG:		Anatomical Site:	
0	19 (38.8)	Cardia	22 (45)
1	24 (49)	Body/pylorus	27 (55)
2	6 (12.2)		
Surgery:		pT Stage:	
Gastrectomy	30 (61.2)	pT1	3 (6.1)
Gastroresection:	19 (38.7)	pT2	5 (10.2)
Billroth I	3 (15.8)	pT2a	1 (2)
Billroth II	16 (84.2)	pT2b	18 (36.7)
		pT3	15 (30.6)
		pT4	7 (14.3)
Lymphadenectomy:		pN Stage:	
D1	13 (26.5)	N0	6 (12.2)
D2	36 (73.5)	N1	19 (38.8)
		N2	15 (30.6)
		N3	9 (18.4)
		pM Stage:	
		Mx	46 (93.9)
		M0	3 (6.1)
		TNM:	
		IB	7 (14.3)
		II	10 (20.4)
		IIIA	17 (34.7)
		IIIB	8 (16.3)
		IV	7 (14.3)

Thirty-six patients (73.5%) received D2 lymph node dissection and 13 patients (26.5%) received D1 dissection. Patients with R1 surgical margins were 16 (32.6%).

Twenty-four patients (49%) received Mcd regimen and 25 patients (51%) received ECF scheme. All patients completed RT as planned: 45 Gy in 33 patients (67.4%); 50.4 Gy in 16 patients (32.6%).

Acute grade 3–4 toxicities were observed in 6 patients (12.1%) (Table 2). No patients required hospital recovery for acute toxicity during adjuvant treatment.

**Table 2:** Toxicity grade sec CTCAE V4.0

	Number of patients (%)		
Toxicity	Grade 2	Grade 3	Grade G4
Gastrointestinal	3 (6)	1 (2)	-
Weight loss	3 (6)	2 (4)	1 (2)
Thrombocytopenia	2 (4)	-	-
Anaemia	2 (4)	1 (2)	-
Mucositis	2 (4)	1 (2)	-
Neurological	2 (4)	-	-
Skin reaction	1 (2)	-	-

Overall, 46 patients (94%) receive full dose of CT. Three patients (6%) were unable to complete CT: 1 patient due to G3 gastrointestinal toxicity and 2 patients due to reduced compliance. No patient had treatment interruption for progressive disease.

### Overall survival and disease-free survival analysis

Overall, median OS and DFS were 46 months and 39 months, respectively. Thirty-one patients (63.3%) had died. The 2-year and 5-year OS rates for the entire population were 65.3% (95% CI 0.503–0.768) and 41.5% (95% CI 0.268–0.545), respectively. The 2-year and 5-year DFS were 59.2% (95% CI 0.442–0.714) and 41.2% (95% 0.271–0.548), respectively. The prognostic analysis is shown in Table 3. No prognostic factors were significantly associated with OS.

**Table 3:** Univariate and multivariate analysis of prognostic factor for overall survival

	Univariate analysis		Multivariate analysis	
Prognostic factor	HR (95% CI)	*p* value	HR (95% CI)	*p* value
Sex (male vs female)	0.59 (0.82–3.42)	0.15	0.55 (0.88–3.79)	0.10
Age (< 65 vs ≥ 65)	1.10 (0.43–1.88)	0.79		
Grading (G1-2 vs G3)	1.59 (0.28–1.41)	0.25	1.81 (0.24–1.27)	0.16
Tumour location (cardia vs body/pylorus)	0.95 (0.52–2.12)	0.89		
Chemotherapy regimen (Mcd vs ECF)	0.81 (0.59–2.54)	0.56		
Lymph node dissection (D1 vs D2)	0.78 (0.54–2.96)	0.57		

Abbreviation: Mcd: Macdonald.

Overall, 30 patients (61.2%) relapsed. Most of these patients (*n* = 28, 93.3%) had distant metastases, in 8 cases (28.6%) associated with loco-regional failures. Only 2 patients (6.7%) had local recurrences and both within the first year after the end of treatment (1-year DFS = 69.4%, 95% CI 0.544–0.803).

### Subgroup survival analysis

**Figure 1:**
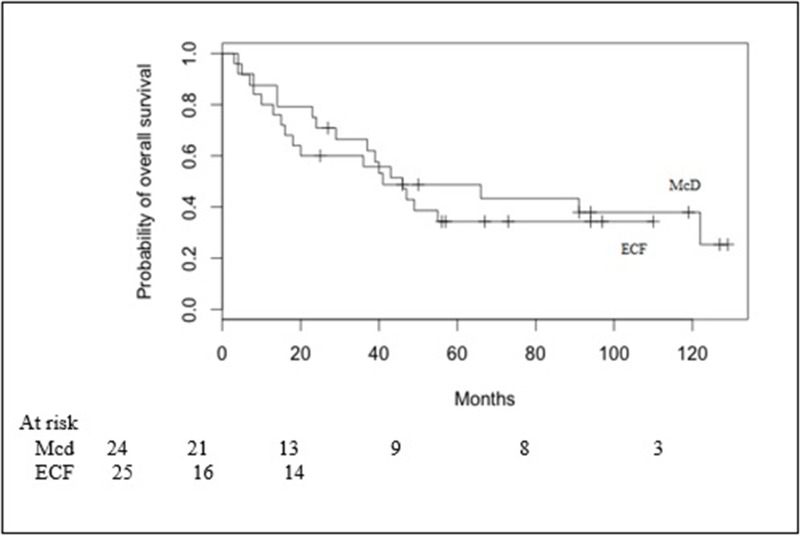
Overall survival in Macdonald (Mcd) patients and ECF patients

The 2-year OS for Mcd group versus ECF group was 70.8% (95% CI 0.484–0.849) versus 60.0% (95% CI 0.384–0.761); the 5-year OS for Mcd group versus ECF group was 48.7% (95% CI 0.276–0.669) versus 34.3% (95% CI 0.166–0.528) (*p* = 0.568) (Figure 1).

**Figure 2:**
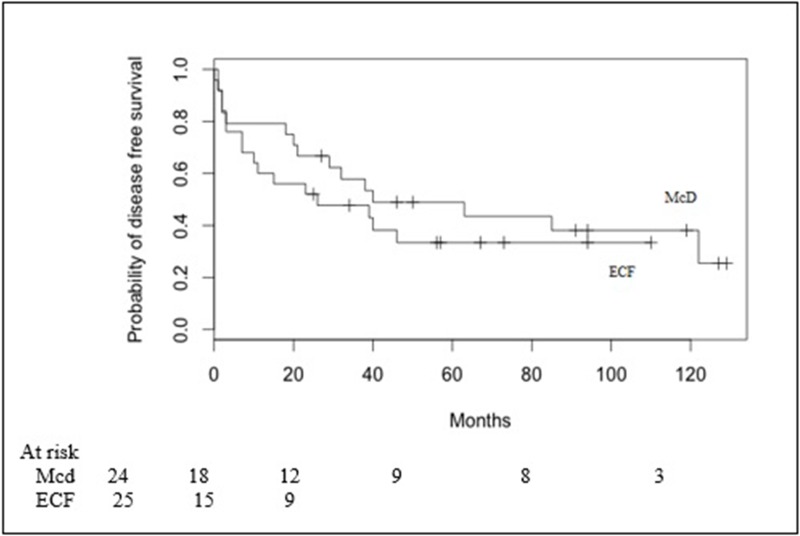
Disease free survival in Macdonald (Mcd) patients and ECF patients

The 2-year and 5-year DFS in Mcd group versus ECF group were 66.7% (95% CI 0.443–0.817) versus 52% (95% CI 0.313–0.692) and 48.9% (95% CI 0.278–0.67) versus 33.4% (95% CI 0.156–0.523) (*p* = 0.404), respectively (Figure 2).

## DISCUSSION

GC is usually diagnosed in locally advanced stage. Prognosis is poor, with 5-year OS of 5–20% [5]. Surgery is considered the current standard recommendation. However, survival rates remain quite low despite tumor curative resection and therefore different adjuvant strategies were extensively tested. At present, no standard CT regimens have been defined and the role of adjuvant RT after an adequate lymph node dissection is unclear.

It is well established that survival outcomes significantly differ between Asian and Western cohort studies [6]. Thus it is difficult to compare their results mainly due to differences in disease natural history, diagnostic approach and treatment strategy.

For instance, Japanese and other Asian surgeons routinely perform a D2 lymph node dissection to remove the nodes along the mains branches of the celiac axis. Whereas the vast majority of Western surgeons perform a D1 dissection, including only lymph nodes close to surgical stomach bed, in order to reduce morbidity and mortality rates.

Both Dutch trial [7] and MRC (Medical Research Council) trial [8] registered high postoperative complications and mortality rates following D2 dissection without a significant improvement in survival outcomes compared to D1 dissection. The update data of Dutch trial showed a lower LR rate and cancer-related deaths in D2 lymphadenectomy [9]. However, it should be emphasized that in more advanced stages even with an adequate lymphadenectomy the results of surgery in Western patients are still unsatisfactory [10]. In this setting of patients, additional treatments should be planned to improve patients' long-term survival.

Based on MAGIC trial results [11], nowadays the neoadjuvant approach is recommended. The aim of neoadjuvant CT (NACT) is to downstage and downsize primary tumor and to treat potential micro-metastasis.

Different studies demonstrated a significant improvement in OS rates of NACT versus surgery alone. Schuhmacher *et al*. [12] reported that NACT improved R0 resection rate without impact on OS rate. Similarly, Stahl *et al*. [13] showed a higher rate of complete responders and Van Hagen *et al*. [14] reported an improved OS. A recent meta-analysis of 1820 patients with advanced GC confirmed NACT survival benefit (OR: 1.32; 95% CI: 1.07–1.64; *p* < 0.01), 3-year PFS (OR: 1.85; 95% CI: 1.39–2.46; *p* < 0.0001), tumor down-staging rate (OR: 1.71; 95% CI: 1.26–2.33; *p* < 0.0006) and R0 resection rate (OR: 1.38; 95% CI: 1.08–1.78; *p* < 0.01) [15].

The aim of this study was to investigate OS, DFS, toxicity rate and treatment compliance in patients with locally advanced GC treated with postoperative CRT. Two CT regimens were analysed: Mcd and ECF. Mcd group achieved higher DFS and OS rates then ECF group.

In the Intergroup trial INT-0116 [1] a significant OS improvement resulted in those patients who received adjuvant CRT versus surgery alone. Authors reported that 19% of patients in the CRT arm had LR versus 29% in the control arm. However, the high LR rate should be related to limited lymph node dissection. Our median OS in Mcd regimen was higher than INT 0116 trial (46 months versus 36 months). It should be noted that 73.5% of our patients underwent D2 dissection versus 10% in the INT 0116 trial. In our study, all patients with D1 dissection developed LR.

In the Korean ARTIST study [16], after gastrectomy with D2 dissection, patients were randomized to adjuvant CT or adjuvant CRT. There were no differences in DFS and OS between the two groups, but a subgroup analysis showed an improvement in DFS in the CRT group (*p* = 0.0365).

The European CRITICS trial [17] evaluated the clinical outcome for adjuvant CT versus adjuvant CRT after 3 cycles of NACT. The results are expected.

Our patients completed planned RT and 94% of patients received CT at full dose. The optimal toxicity rate (mainly gastrointestinal) was probably related to modern RT technique. In fact, since 2004, RT was delivered with 3DRT that allows to conform the radiation dose on the PTV and to reduce the volume and the dose to the surrounding OARs. Whereas, literature data still refers to a 2DRT technique, using two fields AP and PA. Maybe, in our study, premedication to CRT, including metoclopramide, ondansetron and dexamethasone, contributed to lower toxicity rates, too.

In the INT 0116 trial, 36% of patients did not complete the treatment, mainly due to G3-4 toxicities (17% of cases). In a retrospective study of Kundel *et al*. [18] higher toxicity rates were registered: 46.4% of patients presented severe adverse events, 32% were hospitalized and 1.8% died.

In this analysis, sex, age, tumor grading, tumor site, CT regimen and type of lymph node dissection were no significantly associated with OS. However, in several retrospective studies some prognostic factors were demonstrated [19–21]. N-category and N-ratio interaction, perineural invasion and extended resections were prognostic factors for survival in GC patients treated with D2 dissection in a retrospective study by Costa *et al*. [21]. Therefore, it seems that a category of patients may benefit from an intensified therapy. This setting of patients may be treated with NACT, surgery and adjuvant treatment, while other types of patients may benefit from a radical surgery followed by adjuvant therapy.

In conclusion, adjuvant CRT was a feasible strategy in locally advanced GC. It had an acceptable toxicity rate and it was able to increase both DFS and OS, even after extended lymph node dissection. Modern 3DRT reduced toxicity effects.

As a future perspective, it is desirable to find prognostic factors to select patients in order to provide a personalized therapy.
